# Cancer Cured by Electricity

**Published:** 1877-12

**Authors:** 


On the 19th of September, 1877, a gentleman, resident of a western city, called to consult me about a painful tumor located in front of the right ear. The patient is about sixty years old, tall and slender. Having been a quiet and successful business man, of most exemplary life, he has escaped those severer cares which impair the vital power, and favor the development of diseases.

The tumor appeared in May last. When first observed it was the size of a small pea, but grew rapidly in spite of all efforts to arrest its progress. When I first saw it—Sept. 1[illegible]th—it had arrived at the period of ulceration, and a portion of the surface as large as the thumb nail was coated over with blood, mixed with a thin, acrid fluid, which oozed out in sufficient quantity to keep the surface constantly moist. By applying glycerine, and afterward carefully removing this coating, I was able to examine the tumor. It was oval, with jagged edges which were elevated above the center of the tumor, where there was a small pit or indentation, surrounded with irregular cells, giving it a warty appearance. The edges projected over the circumference as if a thread had been drawn tightly around its base. This gave it the appearance of what is termed a "rose cancer." It was evident from the appearance of the edges of the sore, and from other indications, that the disease was extending rapidly. The tumor was larger in diameter than the surface of the denuded portion, for the surrounding parts, to the width of half an inch from the edges of the sore, were swollen and indurated, and the opening into the right ear was nearly closed by the pressure of the tumor upon it.

The patient complained of severe lancinating or shooting pains, and of a burning and "clawing" sensation more unendurable than the sharp pains. These symptoms had been almost constant during the previous thirty days. There was the peculiar tint of the complexion, and the anxious look which denote constitutional disturbance. Had the tumor been in its first stage and confined to narrow limits, its destruction by the application of "chloride of zinc," or "arsenical paste," might have been successfully accomplished. But that treatment would have been extremely hazardous, not only on account of the advanced stage of the disease, but also because of its unfavorable location. In this view of the case, I determined to test the merits of electricity— that mysterious agent, whose curative power is now admitted by all intelligent physicians. In a book entitled "Practical Application of Electricity," by J. H. Rae, M. D., Bible House, New York, I had read with great interest, the report of successful treatment by electricity of an aggravated case of cancer. The patient was the Hon. Thomas T. Davis, formerly a member ot Congress from this State. The facts are authenticated by some of the most prominent physicians of this city. I advised that Dr. Rae should be consulted.

Another physician of education and great experience was called in, and we all agreed that the disease was cancer. Of many cases successfully treated by Dr. Rae, this was the first where ulceration had commenced. For this reason I was quite disposed in my own mind, to regard the treatment as a "forlorn hope." On the other hand, the Doctor seemed to have entire confidence in the power of electricity to eradicate the disease from the system.

To make this article of practical value to sufferers from this most unfortunate malady, it is necessary to state clearly all the essential facts connected with the case. The electrical instrument employed by Dr. Rae is his own invention. It has three distinct currents; the "direct," the "induced," and the "superinduced." He claims that each of these currents are required in the course of the treatment. His "electrodes" are of solid silver, which he claims is important. The greatest care is exercised in the arrangement and adjustment of the instrument. The battery is closely looked after, and any defect is quickly detected and remedied. Everything being ready, the treatment was commenced September 20, 1877. The application of electricity to the tumor was attended by little or no pain, and was continued not more than fifteen minutes; yet it at once produced a marked change in the symptoms. The shooting pains and the burning sensation disappeared, not to return during the treatment In their stead was a dull pain or aching, which contiuued constantly until after the treatment the next day, when the pain was less, with occasional intermissions. September 22--after treatment to-day, the treatment was suspended for three days. September 25—treatment resumed and continued daily till September 29, when general treatment by electricity was employed in addition to the local application. The pain during the last two days has been slight, and only at long intervals. The ulcerated surface has sensibly contracted, and the adjacent swelling and induration have diminished. Treatment suspended for one week. Oct 6—Treatment re-commenced, and continued daily. Oct. 15—The swelling and induration about the sore have entirely disappeared. The opening into the right ear is restored to its natural size and condition All pain has ceased, and the tumor seems to be dead. The granulations on the edges of the skin surrounding it have assumed a healthy appearance. In order to prevent absorption of the dead tumor, I have commenced cutting it down with nitrate of silver, applying it twice a day to remove it rapidly.

Oct. 22—All signs of the tumor have disappeared, leaving the surface of the sore—which has now contracted to the size of a split pea—in a healthy condition. Oct. 23—Took off the small piece of court plaster, and found the skin was forming over the remains of the sore. Patient left for home. Oct. 31—A letter from my patient, dated Oct. 29, says: "Sore entirely healed; am all right as yet." This is expressed with the caution characteristic of the man. From the manner of the cure, which was evidently effected by a destruction of the germs of the disease in the system, I do not think it probable that the disease will return. The name and address of my patient will be given to sufferers from cancer, if desired, but to such only.

To those who believe that cancer is essentially an hereditary disease to be found only in certain families, an additional reason, if any were needed for the belief that this was cancer, may be found in the fact that a sister of my patient has recently submitted to a surgical operation for the removal of a cancer.

December 7th, a letter just received from my patient dated December 4th—six weeks after the cure was completed—states that there is not the least indication of a return of the disease, and that his general health is better than it has been for years. He still uses the "electrical foot bath" once a week as a tonic, and to eliminate from the system any vestiges of the disease that might remain.

I have thus endeavored to give a faithful account of the treatment of a case which has greatly interested me, and the favorable result of which has been the most agreeable surprise of my medical experience. I have watched the ravages of this sad disease with especial interest from my student days, when under the tuition of that eminent surgeon—Professor Frank H. Hamilton of this city. Nine years ago I visited the crowd of cancer patients who were lured to the worthless springs of Sheldon, Vermont, and during a summer pursued my investigations there. I have studied the disease in all its forms, during three years in the hospitals of Paris, under the instructions of Velpeau and Nelaton, and Joubert. I have assisted at hundreds of operations where the results hoped for seemed hardly sufficient to compensate for the sufferings endured; but I never before saw an effort made to cure the disease by electrical treatment, which is attended by neither pain nor inconvenience, and which seems to be radical and permanent. It is, perhaps, too soon to proclaim that electricity is the long sought specific for the cure of cancer. In my opinion it is more likely to prove such than any other remedy yet devised.

Electricity has long been regarded as an important therapeutic agent by the medical profession. Nearly all of our New York physicians prescribe it in cases of tumors, scrofulous affections, and nervous diseases. I am satisfied that the list of diseases to the cure of which it is adapted may be greatly extended. I have seen it employed successfully in the treatment of rheumatism, sciatica, lumbago, cystitis, and in various forms of kidney troubles, when other remedies had proved unavailing. Although an agent not to be trifled with, or ignorantly employed, in skillful hands its use is not attended with risk, pain, or inconvenience, while its tonic effect in the system cannot fail to be of benefit in every instance where debility exists from any cause whatever.

